# Reprogrammed astrocytes display higher neurogenic competence, migration ability and cell death resistance than reprogrammed fibroblasts

**DOI:** 10.1186/s40035-020-0184-6

**Published:** 2020-02-08

**Authors:** Xiaohuan Xia, Chunhong Li, Yi Wang, Xiaobei Deng, Yizhao Ma, Lu Ding, Jialin Zheng

**Affiliations:** 1 Center for Translational Neurodegeneration and Regenerative Therapy, Shanghai Tenth People’s Hospital affiliated to Tongji University School of Medicine, Shanghai, 200072 China; 2 Collaborative Innovation Center for Brain Science, Tongji University, Shanghai, 200092 China; 3 Departments of Pharmacology and Experimental Neuroscience, University of Nebraska Medical Center, Omaha, NE 68198-5930 USA; 4 Department of Pathology and Microbiology, University of Nebraska Medical Center,, Omaha, NE 68198-5930 USA

**Keywords:** Reprogramming, Astrocyte, Fibroblast, Induced neural progenitor cells, TGFβ signaling, Neurogenesis, Proliferation, Migration, Survival

## Abstract

The direct reprogramming of somatic cells into induced neural progenitor cells (iNPCs) has been envisioned as a promising approach to overcome ethical and clinical issues of pluripotent stem cell transplantation. We previously reported that astrocyte-derived induced pluripotent stem cells (iPSCs) have more tendencies for neuronal differentiation than fibroblast-derived iPSCs. However, the differences of neurogenic potential between astrocyte-derived iNPCs (AiNPCs) and iNPCs from non-neural origins, such as fibroblast-derived iNPCs (FiNPCs), and the underlying mechanisms remain unclear. Our results suggested that AiNPCs exhibited higher differentiation efficiency, mobility and survival capacities, compared to FiNPCs. The whole transcriptome analysis revealed higher activities of TGFβ signaling in AiNPCs, versus FiNPCs, following a similar trend between astrocytes and fibroblasts. The higher neurogenic competence, migration ability, and cell death resistance of AiNPCs could be abrogated using TGFβ signaling inhibitor LY2157299. Hence, our study demonstrates the difference between iNPCs generated from neural and non-neural cells, together with the underlying mechanisms, which, provides valuable information for donor cell selection in the reprogramming approach.

## Background

Neuronal loss is a key pathological attribute of neurodegenerative diseases (ND), such as Alzheimer’s disease (AD) [1] and Parkinson’s disease (PD) [2]. Due to the failure of clinical trials aiming to eliminate classical disease-associated molecules (e.g. Aβ) [3], cell transplantation, which may replace degenerating neurons or provide protective microenvironments, is considered as a promising therapeutic strategy for ND treatment [4, 5]. The direct conversion of somatic cells into self-renewable and lineage-restricted induced neural progenitor cells (iNPCs) overcomes various concerns for the clinical applications of embryonic stem cells (ESCs) [6] and induced pluripotent stem cells (iPSCs) [7] including autogenous immune response of their host, ethical and religious concerns, and high risk of tumor formation [8–12]. Therefore, iNPCs seem like a better option for transplantation.

To date, multiple types of somatic cells have been used for generating iNPCs [8, 10, 13]. Donor cells influence the genetic and epigenetic patterns of reprogrammed cells, leading to diverse differentiation potential of the latter [14–17]. However, whether the central nervous system (CNS) cell-derived iNPCs and non-neural cell-derived ones exhibit different neurogenic potential remains unclear. Here, we reprogrammed fibroblasts into iNPCs through ectopic expressing transcription factors Sox2, Brn2, and Foxg1, the same method we previously used for astrocyte reprogramming [10]. We found that astrocyte-derived iNPCs (AiNPCs) exhibited higher differentiation efficiency, mobility and cell death resistance than fibroblast-derived iNPCs (FiNPCs). We further observed the activation of TGFβ signaling in AiNPCs, which is likely due to inheriting the active status of TGFβ signaling in astrocytes. The inhibition of TGFβ signaling reduced the differentiation, migration, and survival capacities of AiNPCs. Together, our data extend our understanding on somatic reprogramming, providing valuable information for the development of cell-based therapies in ND.

## Methods

### Isolation of mouse fibroblasts

Mouse fibroblasts were derived from Nestin-EGFP transgenic mice embryos at embryonic day 13.5–14.5 (E13.5-E14.5) as previously described [18]. Briefly, all the internal organs, head and spinal cord were removed from embryos. The remaining skin tissues were washed twice with PBS, and dissociated with 0.25% trypsin-EDTA solution. Mouse fibroblasts were cultured in high glucose medium supplemented with 10% FBS, 1% non-essential amino acid (non-AA), 100 U/ml penicillin, 100 μg/ml streptomycin at 37 °C in a 5% CO_2_ humidified atmosphere.

### Isolation and enrichment of NPCs

Control neural progenitor cells (NPCs) used in this study were generated from E13.5-E14.5 mouse cortices as previously described [10]. Briefly, the cortices were dissected out and dissociated into single cells by physical triturating with a 1 ml pipette. Filtered cells were seeded into 100 mm non-coated Petri dishes (Fisher) at a density of 2 × 10^5^ cells/ml in 10 ml of NPC favoring medium (NPCM) containing NeuroCult® NSC Basal Medium (Stem Cell Technologies), NeuroCult® NSC Proliferation Supplements (Stem Cell Technologies), 20 ng/ml bFGF (BioWalkersville), 20 ng/ml EGF (BioWalkersville), 100 U/ml penicillin (Gibco) and 100 μg/ml streptomycin (Gibco) for primary neurosphere formation. Culture medium was replaced every two days. Neurospheres were passaged every 3–4 days when they reached 150 μm in diameter.

### Retroviral vectors and retrovirus preparation

Plasmid encoding mouse Sox2 was purchased from Addgene (Plasmid #13367). Mouse Foxg1 (restriction enzymes: BamHI and XhoI) and Brn2 (restriction enzymes: BamHI and XhoI) were amplified from mouse control NPCs cDNA library. Each gene was individually cloned into pMXs-retroviral vectors (Cell Biolabs, RTV-010).

Retroviruses (pMXs) were generated with Plat-E packaging cells as previously described [10]. Briefly, Plat-E cells were seeded at 1 × 10^6^ cells in 100-mm cell culture dish for per virus (pMXs empty vector, Sox2, Brn2, Foxg1)-four dishes in total. After 1 day, the retroviral vectors were packaged by transfection reagent lipofectamine LTX reagent (Invitrogen, A12621). After 48 h of transfection, supernatant with viral particles was collected and filtered through a syringe attached to a 0.45-μm filter. Retroviruses were harvested though centrifugation at 15,000 × rpm for 1.5 h at 4 °C.

### Reprogramming of mouse fibroblasts

The direct reprogramming of fibroblasts into iNPCs was performed in the same way for the reprogramming of astrocytes as previously described [8, 10]. Briefly, mouse fibroblasts were incubated in the mixed virus-containing supernatants overnight. 10 μg/mL polybrene (Millipore) was added to facilitate virus transfection. Infected fibroblasts were cultured in NPCM 1 day after the second infection. NPCM was replaced every two days. Twenty-eight days after retroviral transduction, Nestin-EGFP^+^ colonies were formed, manually picked and suspended into single cells to generate neurospheres. Floating primary neurospheres were collected after culturing for 4-6 days and re-plated into Poly-D-Lysine/Fibronectin-coated 6-well plates. Cells were collected after reaching 80% confluency and re-suspended into single cells for a second round of neurosphere formation. After 3 rounds of selection and enrichment, cells were collected for NPC characterization.

### Neurosphere formation assay

Neurosphere formation (self-renewal) assay was performed by suspending 1 × 10^4^ cells with NPCM in each well of 6-well plates. Fresh medium was added into the suspension culture every other day. The size and numbers of neurospheres were quantified at culture day 4 under the bright field of a microscope.

### Wound healing assay

FiNPCs and AiNPCs were plated in matrigel-coated 6-well plates and grown until 80% confluent. Wound was made using 200 μm pipette tip. Cells were washed with PBS twice and incubated at 37 °C for 24 h in NPCM. The external surface of each well was marked for the observations and microscopy of identical fields at different times. Images were captured using an EVOS™ XL Imaging System (Thermo Fisher Scientific). For quantification, cells which migrated into scratched region were counted from 10 fields per well.

### Transwell migration assay

Migration assays were carried out as previously described [19]. Briefly, 24-well transwell using polycarbonate membranes with 8-μm pores (Corning Costar) was coated by matrigel (BD bioscience). iNPCs at a density of 5 × 10^5^ cells/ml in 100 μl of NPCM were placed in the upper chamber of the transwell assembly. The lower chamber contained 500 μl of NPCM. After 12 h, the membrane of the transwell inserts was fixed with 4% paraformaldehyde (PFA) in PBS, and non-migrating cells on the top of the membrane were removed with a cotton swab. Cells that migrated to the bottom of the membrane were stained with DAPI using VectaShield (Vector Laboratories). Images were captured using a Zeiss AX10 fluorescence microscope accompanied with ZEN 2.3 (blue edition) software. For quantification, DAPI labeled cells were counted from 10 random fields per insert.

### RNA isolation and quantitative polymerase chain reaction (qPCR) analysis

Total RNA was isolated by RNeasy mini kit (Qiagen) according to the manufacturer’s instructions. DNase I digestion kit (Qiagen) was used to remove genomic DNA. cDNA was synthesized from mRNA using the SuperScript III reverse transcriptase kit (ThermoFisher). RNase inhibitor was used to prevent RNA degradation during reverse transcription. Transcripts were amplified using gene-specific primer (Additional file 1: Table S1) and SYBR Green PCR Master Mix (Applied Biosystems) with Lightcycler® 96 PCR system (Roche). All mRNA expression levels were normalized to housekeeping gene GAPDH and calibrated on the control cells specified in each experiment.

### Immunocytochemistry

Cells were fixed in 4% PFA (Sigma) for 15 min at room temperature (RT), rinsed 3 times with PBS, and then incubated with permeabilizing/blocking buffer containing 5% normal goat serum (Vector Laboratories) and 0.4% Triton X-100 (Bio-Rad) in PBS for 30 min at RT. Cells were incubated with primary antibody solutions (Additional file 1: Table S2) overnight at 4 °C. The following day cells were washed 3 times with PBS and incubated with secondary antibodies (Molecular Probes) for 2 h at RT. Cells were counterstained with VectaShield (Vector Laboratories). IgG control was used as negative controls. Images were captured using a Zeiss AX10 fluorescence microscope accompanied with ZEN 2.3 (blue edition) software. For quantification, cell type-specific antigen positive cells were counted from 15 random fields per group in three cover slips (5 fields each).

### Neuronal differentiation

iNPCs were plated on matrigel-coated 24-well plates (1 × 10^5^ cells/well) or 6-well plates (5 × 10^5^ cells/well) and cultured in basic differentiation medium contained DMEM/F12 (Gibco), 2% Knockout Serum (Gibco), 1 × N2 (Invitrogen), 1 × B27 (Invitrogen), 2 mM L-glutamine (Gibco), 100 U/ml penicillin (Gibco) and 100 μg/ml streptomycin (Gibco) to facilitate differentiation. Differentiation was terminated 7 days after plating. For oligodendrocyte differentiation, iNPCs were cultivated in DMEM/F12, supplemented with 1 × N2 (Invitrogen), 10 ng/ml PDGF (R&D systems), 10 ng/ml bFGF (BioWalkersville), 2 mM L-glutamine (Gibco), and 10 mM forskolin (R&D systems) for 4 days. Afterwards, PDGF and forskolin were replaced by 30 ng/ml 3, 3, 5-triiodothyronine (T3) hormone (Sigma-Aldrich) and 200 mM ascorbic acid (Sigma-Aldrich) for another 7 days.

### TUNEL assay

iNPCs in proliferation and differentiation conditions were stained with TUNEL assay (Roche Diagnostics). All experiment procedures were performed following manufacturer’s instructions. TUNEL^+^ cells and total cells were counted after acquiring random images from immunostained fields using a Zeiss AX10 fluorescence microscope accompanied with ZEN 2.3 (blue edition) software. A minimum of 10 fields was counted for each treatment condition.

### RNA-seq analysis

Total RNA was extracted from iNPCs using RNeasy mini kit (Qiagen). Sample processing was carried out by Novogene Corporation using the Illumina HiSeq platform. Sequencing libraries were generated using NEBNext® UltraTM RNA Library Prep Kit for Illumina® following manufacturer’s instructions and index codes were added to attribute sequences to each sample. The clustering of the index-coded samples was performed on a cBot Cluster Generation System using TruSeq PE Cluster Kit v3-cBot-HS (Illumia) following manufacturer’s instructions. The prepared libraries were sequenced and 125 bp/150 bp paired-end reads were generated. Index of the reference genome was built paired-end clean reads were aligned to the reference genome using Hisat2 v2.0.5. RNA-seq reads counting was done using featureCounts v1.5.0-p3 and fragments per kilobase of transcript per million fragments mapped (FPKM) of each gene was calculated based on the length of the gene and reads count mapped to this gene. Differential expression analysis was performed using the DESeq2 R package. *P*-values and q-values were adjusted using the Benjamini and Hochberg’s approach for controlling the false discovery rate. Genes with q-value < 0.05 found were assigned as differentially expressed. Gene Ontology (GO) and Kyoto Encyclopedia of Genes and Genomes (KEGG) pathway analyses of differentially expressed genes were carried out using DAVID Bioinformatics Resources 6.8 (https://david.ncifcrf.gov/home.jsp). *Mus musculus* genome data and hypergeometric statistical method were used for KEGG enrichment analyses. Benjamini & Hochberg multiple test adjustment was used to adjust *P*-value of analysis: P-value < 0.05 was considered a significant enriched pathway.

### BiSulfite amplicon sequencing

Genomic DNA was isolated using DNeasy Blood & Tissue Kit (Qiagen). Bisulfite-conversion-based Methylation PCR Primers were design using MethPrimer (http://www.urogene.org/methprimer/index.html). Bisulfite treatment was carried out using 500 ng of DNA and the EZ DNA Methylation-Direct kit (Zymo Research). This process deaminated unmethylated cytosine (C) residues to uracil (U) leaving methylated cytosine (^m^C) residues unchanged. The PCR reactions were performed in a total volume of 25 μl for 45 cycles using 0.25 μl Takara Ex Taq HS, 2.5 μl 10 × Ex Taq buffer, 2 μl dNTP mixture (2.5 mM each), 0.5 μl forward primer (10 μM), 0.5 μl Reverse Primer (10 μM) under the following conditions: 95 °C for 15 s, 55 °C for 20 s and 72 °C for 40 s. Bisulfite-treated DNA was used as template. All PCR products were electrophoresed, collected, and purified by GeneJET Gel Extraction Kit (Thermo). Library was built using VAHTSTM Turbo DNA Library Prep Kit for Illumina and methylation percentage of each CpG was determined by Illumina Miseq system, according to recommendations from the manufacturer.

### Statistical analyses

Data from two groups were compared with two-tailed, paired or unpaired Student’s *t* tests (Graphpad Prism 5.0 software). Data were shown as mean ± s.d., and significance was determined as *P* < 0.05.

## Results

### AiNPCs have higher neurogenic, invasive and survival potential than FiNPCs

**Fig. 1:**
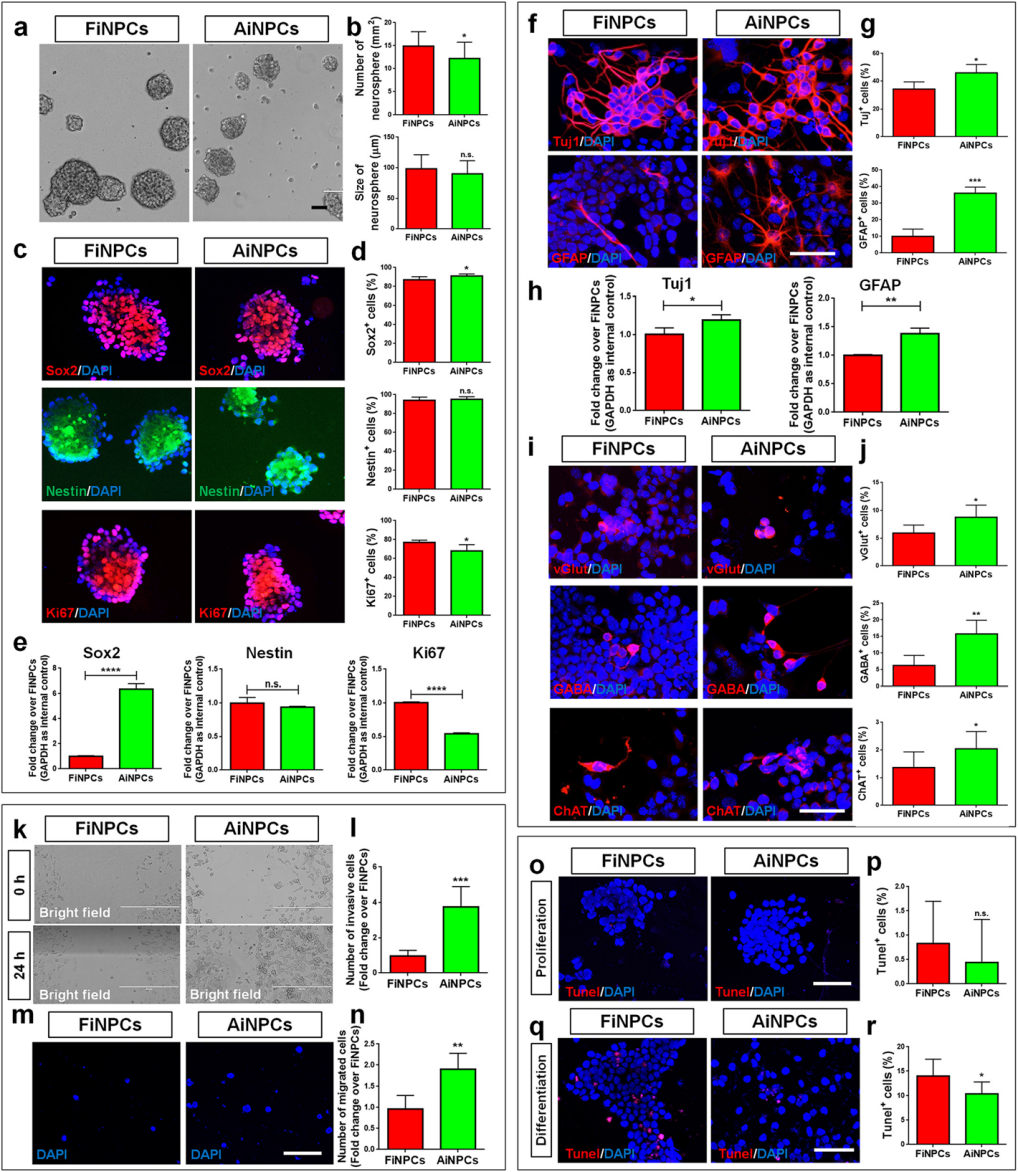
The comparison of FiNPCs and AiNPCs **a** Photographs of identical fields of neurospheres were taken for FiNPCs and AiNPCs after cultured in proliferation conditions for 3 days. **b** The number and size of neurospheres in each field were measured. **c** Cells expressing Sox2, Nestin or Ki67 specific immunoreactivities in the FiNPCs and AiNPCs groups. **d** The proportions of Sox2^+^, Nestin^+^, or Ki67^+^ cells in each field were counted. **e** The transcript expression of *Sox2*, *Nestin* and *Ki67* was determined by qPCR analysis. **f** Cells expressing Tuj1 or GFAP specific immunoreactivities in the FiNPCs and AiNPCs groups after cultured in differentiation conditions for 3 days. **g** The proportions of cells expressing Tuj1 or GFAP in each field were counted. **h** The transcript expression of *Tuj1* and *GFAP* was determined by qPCR analysis. **i** Cells expressing vGlut, GABA or ChAT specific immunoreactivities in the FiNPCs and AiNPCs groups after cultured in differentiation conditions for 7 days. **j** The proportions of cells expressing vGlut, GABA or ChAT in each field were counted. **k** Photographs of identical fields of cells were taken for the FiNPCs and AiNPCs groups at 0 h and 24 h in wound healing assay. **l** The total number of invading cells of each field was counted and represented in fold change. **m** Photographs of identical fields of DAPI labeled cells were taken for the FiNPCs and AiNPCs groups after cultured in transwells for 12 h. **n** The total number of migrated cells of each field was counted and represented in fold change. **o, q** Photographs of identical fields of TUNEL^+^ cells were taken for the FiNPCs and AiNPCs groups after cultured in proliferation **o** and differentiation **q** conditions. **p, r** The total number of TUNEL^+^ cells in proliferation **p** and differentiation **r** conditions was counted and represented in proportions. Scale bars represent 50 μm (**a, c, f, i, o, q**), 400 μm (**k**), and 100 μm (**m**). Data were normalized to GAPDH and presented as fold change. Error bars denote s.d. from triplicate measurements. *P < 0.05, **P < 0.01, ***P < 0.001, and ***P < 0.0001 by two-tailed *t* test (*n* = 3).

Similar to the generation of AiNPCs [10], fibroblasts were reprogrammed into FiNPCs by overexpressing Sox2, Brn2, and Foxg1 (Additional file 1: Figure S1A). FiNPCs exhibit high proliferative and differentiation capacities in defined conditions, ascertained by expressing proliferative and neural cell markers, respectively (Additional file 1: Figure S1B-F). To identify the differences between non neural- and neural-derived iNPCs, we then examined the proliferative, differentiation, invasive, and survival capacities of FiNPCs and AiNPCs. In proliferation conditions, AiNPCs generated fewer neurospheres (Fig. 1a, b) with less Ki67^+^ cells (Fig. 1c, d) and lower expression of *Ki67* (Fig. 1e) than FiNPCs, suggesting that FiNPCs may have stronger proliferative capacity than AiNPCs. Additionally, though there is no difference of Nestin expression between two iNPC lines, immunocytochemical and qPCR analyses demonstrated higher levels of Sox2 proteins and transcripts, respectively, in AiNPCs versus FiNPCs, suggesting AiNPCs may have higher neural properties (Fig. 1c-e). In differentiation conditions (3 days), AiNPCs generated larger proportions of Tuj1^+^ and GFAP^+^ cells, accompanied with higher *Tuj1* and *GFAP* expression, than FiNPCs (Fig. 1f-h). Besides, we found more glutamatergic, GABAergic and cholinergic neurons differentiated from AiNPCs than FiNPCs in extended culture (7 days), indicating higher efficiency of AiNPCs in generating both glial and neuronal cells (Fig. 1i, j). Furthermore, the wound healing assay revealed that, after 24 h, more AiNPCs migrated into an equivalently sized gap than FiNPCs, which was corroborated by the transwell migration assay, suggesting a higher motility of AiNPCs (Fig. 1k-n). Lastly, TUNEL assay showed that both FiNPCs and AiNPCs exhibited very low apoptosis rate (~ 0.5%) in proliferation conditions (Fig. 1o, p). No significant difference was observed between proliferating AiNPCs and FiNPCs. But less TUNEL^+^ cells were observed in differentiated AiNPCs versus differentiated FiNPCs, suggesting that AiNPCs might be more resistant to cell death in neurogenesis [20, 21] (Fig. 1q, r). Hence, AiNPCs exhibited higher neurogenic, invasive, and survival potential than FiNPCs, implying astrocytes as a better donor cell type for iNPCs.

### AiNPCs exhibit higher TGFβ activity than FiNPCs

**Fig. 2:**
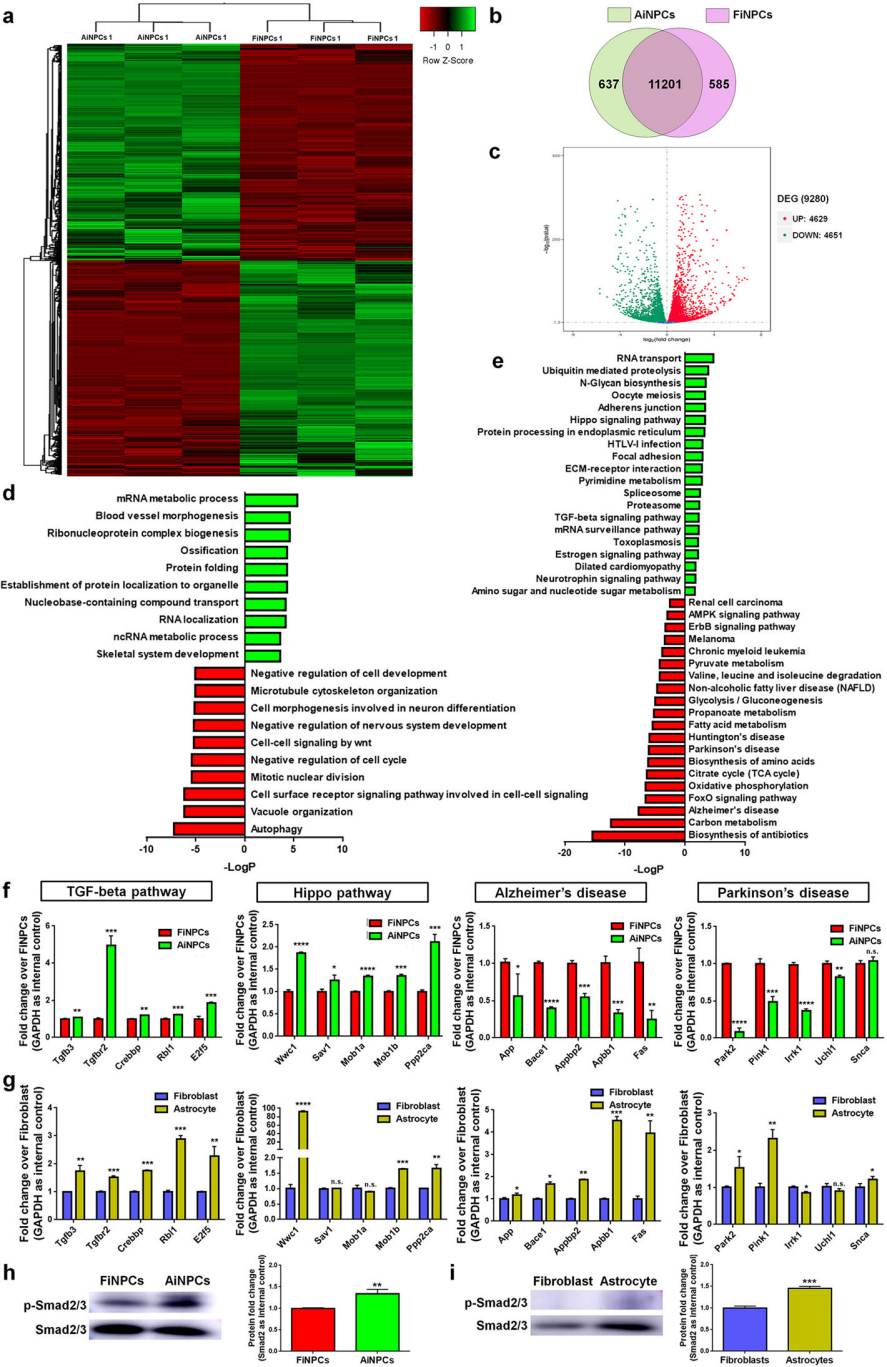
TGFβ signaling displays higher activity in AiNPCs than FiNPCs **a** Hierarchical cluster analysis of differentially expressed genes (DEG) in AiNPCs and FiNPCs. **b** Venn diagram represents the numbers of genes expressed in AiNPCs (yellow) and FiNPCs (pink). **c** Volcano plot upregulated represents the up-regulated (red) and down-regulated (green) genes in the AiNPCs group versus the FiNPCs group. **d** Mapping of the subtracted DEG on GO analysis identified top 10 activated (green) or inhibited (red) biological processes in AiNPCs versus FiNPCs. **e** Mapping of the subtracted DEG on KEGG analysis identified top 20 activated (green) or inhibited (red) signaling in AiNPCs versus FiNPCs. **f, g** qPCR analysis of the subtracted differentially expressed genes (DEG) in TGFβ signaling, Hippo signaling, AD-related signaling, and PD-related signaling in AiNPCs & FiNPCs **f**, and astrocytes & fibroblasts **g**. **h** The phosphorylation levels of Smad2 in FiNPCs and AiNPCs were determined by western blot. **i** The phosphorylation levels of Smad2 in fibroblasts and astrocytes were determined by western blot. The quantification results were given on the right panel. Data were normalized to GAPDH and presented as fold change. Error bars denote s.d. from triplicate measurements. *P < 0.05, **P < 0.01, ***P < 0.00, and ***P < 0.0001 by two-tailed *t* test (*n* = 3).

To understand the potential mechanisms underlying the differences between FiNPCs and AiNPCs, we examined the global gene expression profiles of AiNPCs and FiNPCs (Fig. 2a). The RNA-seq results revealed that 90% genes were commonly expressed in both cell lines, among them, 4629 were up-regulated and 4651 were down-regulated in AiNPCs, compared to FiNPCs (Fig. 2b, c). GO analysis revealed that genes abundantly expressed in FiNPCs were enriched in “Negative regulation of nervous system development” and “Negative regulation of cell development” terms, which may explain the lower differentiation efficiency of FiNPCs than AiNPCs (Fig. 2d). KEGG analysis suggested that genes highly expressed in AiNPCs were enriched in neurogenesis-related signaling, such as Hippo and TGFβ signaling, and genes abundantly expressed in FiNPCs were enriched in ND-related signaling (Fig. 2e). The differentially activated signaling in AiNPCs (TGFβ & Hippo) and FiNPCs (AD & PD) were confirmed qPCR analysis (Fig. 2f). To examine whether iNPCs inherit the genetic and epigenetic signatures from their donors, we tested the activities of these signaling in astrocytes and fibroblast. qPCR analysis revealed that the expression of all tested TGFβ signaling-related transcripts was promoted in astrocytes, versus fibroblasts (Fig. 2g). In contrast, only a subset of Hippo signaling-related transcripts exhibited higher expression levels in astrocytes, compared to fibroblasts. Moreover, the expression of AD- & PD-related transcripts was either higher or no significant difference in astrocytes, versus fibroblast, which did not match with the trends that we observed between AiNPCs and FiNPCs. The western blot analysis also demonstrated higher levels of p-Smad2, the activated form of TGFβ signaling downstream factor Smad2, in AiNPCs and astrocyte, versus FiNPCs and fibroblast, respectively, confirming the selective activation of TGFβ signaling in AiNPCs and their origin cells (Fig. 2h, i). To determine whether the reprogrammed somatic cells persist the epigenetic “memory”, we examined the methylation status of the promoter regions of *Tgfb3* and *Tgfbr2* in FiNPCs, AiNPCs, fibroblasts and astrocytes using BiSulfite Amplicon Sequencing (Additional file 1: Figure S3). Results showed that the promoter regions of *Tgfb3* and *Tgfbr2* were highly demethylated and methylated, respectively, in all tested cell lines. No difference in methylation ratio of *Tgfbr2* promoter was observed among these cell lines, while the *Tgfb3* promoter displayed a slightly but significantly lower methylation status in AiNPCs and astrocytes, compared with FiNPCs and fibroblasts, respectively. It indicated that the higher TGFβ signaling activities in AiNPCs may not be due to the inheritance of the methylation signature from astrocytes, although the biological consequence of slight reduction of the *Tgfb3* promoter CpG methylation in AiNPCs requires further verification. Thus, our results suggest TGFβ signaling is more activated in AiNPCs than FiNPCs.

### TGFβ signaling mediates the neurogenic, invasive and survival potential of iNPCs

**Fig. 3:**
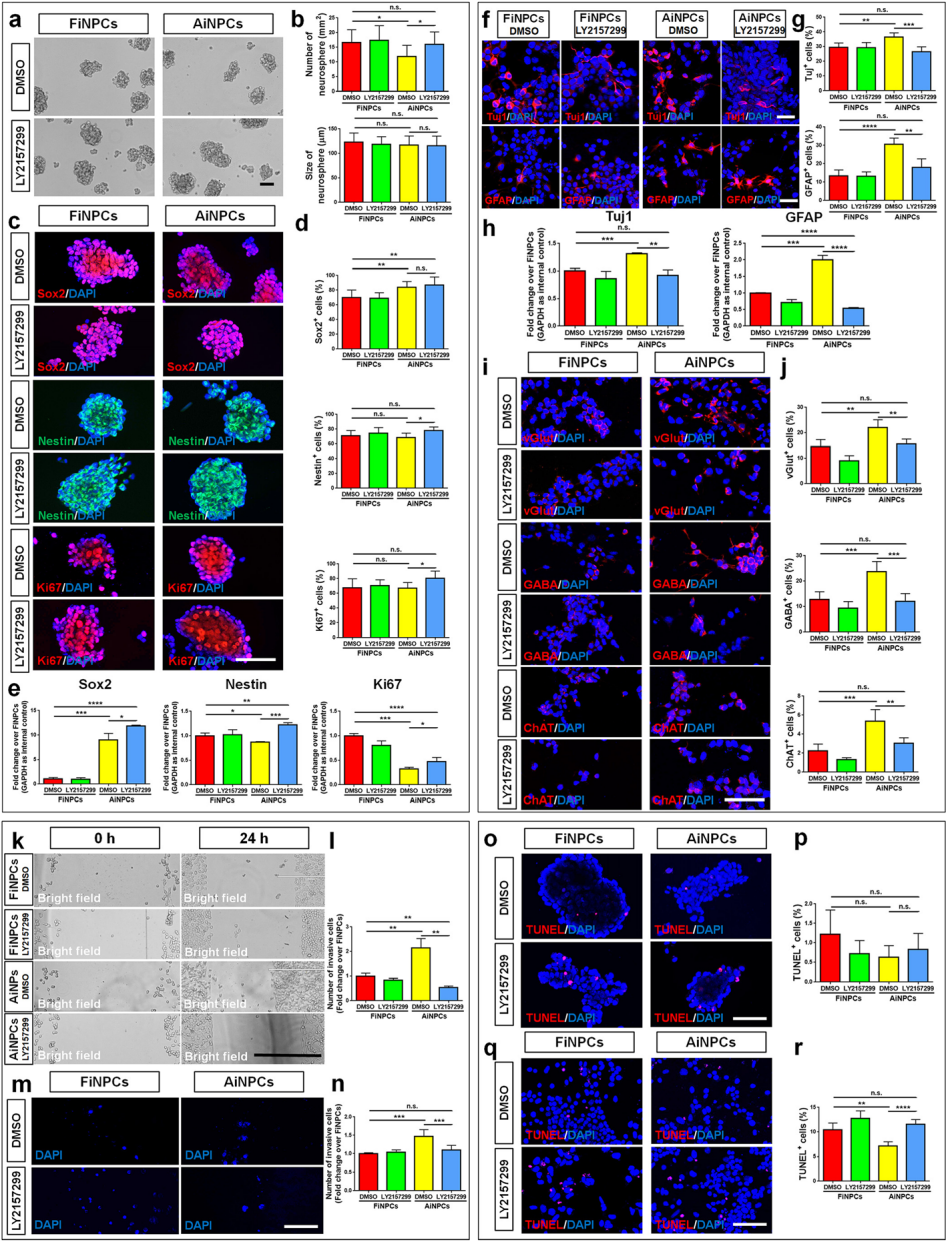
TGFβ signaling mediates neurogenic, migration, and survival capacity of iNPCs. **a** Photographs of identical fields of neurospheres were taken for FiNPCs and AiNPCs in proliferation conditions. **b** The number and size of neurospheres in each field were measured. **c** Cells expressing Sox2, Nestin or Ki67 specific immunoreactivities in FiNPCs and AiNPCs. **d** The proportions of Sox2^+^, Nestin^+^, or Ki67^+^ cells in each field were counted. **e** The transcript expression of *Sox2*, *Nestin* and *Ki67* was determined by qPCR analysis. **f** Cells expressing Tuj1 and GFAP specific immunoreactivities in FiNPCs and AiNPCs in differentiation conditions. **g** The proportions of cells expressing Tuj1 or GFAP in each field were counted. **h** The transcript expression of *Tuj1* and *GFAP* was determined by qPCR analysis. **i** Cells expressing vGlut, GABA or ChAT specific immunoreactivities in FiNPCs and AiNPCs in extended culture. **j** The proportions of cells expressing vGlut, GABA or ChAT in each field were counted. **k** Photographs of identical fields of cells were taken for the FiNPCs and AiNPCs groups at 0 h and 24 h in wound healing assay. **l** The total number of invading cells of each field was counted and represented in fold change. **m** Photographs of identical fields of DAPI labeled cells were taken for FiNPCs and AiNPCs in transwells for 12 h. **n** The total number of migrated cells of each field was counted and represented in fold change. **o, q** Photographs of identical fields of TUNEL^+^ cells were taken for FiNPCs and AiNPCs in proliferation **o** and differentiation **q** conditions. **p, r** The total number of TUNEL^+^ cells in proliferation **p** and differentiation **r** conditions was counted and represented in proportions. Scale bars represent 100 μm (**a, m**), 50 μm (**c, f, i, o, q**), and 400 μm (**k**). Data were normalized to GAPDH and presented as fold change. Error bars denote s.d. from triplicate measurements. *P < 0.05, **P < 0.01, ***P < 0.001, and ***P < 0.0001 by two-tailed *t* test (n = 3).

To examine whether TGFβ signaling mediates the difference between FiNPCs and AiNPCs, we treated FiNPCs and AiNPCs with LY2157299, a TGFβ receptor inhibitor. The inhibition efficiency was validated by western blot (Additional file 1: Figure S2). In proliferation conditions, LY2157299 treatment enhanced the proliferative capacity of AiNPCs, ascertained by the increase of neurosphere numbers (Fig. 3a, b), increase of Ki67^+^ cell proportion (Fig. 3c, d), and elevation of *Ki67* transcript levels (Fig. 3e). Besides, LY2157299 may also increase the NPC phenotype maintenance of AiNPCs as both of the proportion of Nestin^+^ cells and the transcript levels of Nestin and Sox2 increased in LY2157299-treated AiNPCs versus LY2157299-treated FiNPCs (Fig. 3c-e). Under differentiation conditions, LY2157299 treatment reduced the proportions of both Tuj1^+^ and GFAP^+^ cells (Fig. 3f, g) and decreased *Tuj1* and *GFAP* expression (Fig. 3h) in AiNPCs. Besides, the generation of glutamatergic, GABAergic and cholinergic neurons was equally repressed in AiNPCs when TGFβ signaling was repressed (Fig. 3i, j). Moreover, wound healing and transwell assays showed that the higher invasive potential of AiNPCs could be abrogated by LY2157299 treatment (Fig. 3k-n). Lastly, LY2157299 treatment significantly increased the proportions of apoptotic TUNEL^+^ cells under differentiation conditions, suggesting the involvement of TGFβ signaling in maintaining the cell death resistance of iNPCs (Fig. 3o-r). Thus, our results suggest TGFβ signaling plays a key role in mediating the neurogenic, invasive and survival capacities of iNPCs.

## Discussion

The tissue origins have emerged as a key factor in determining the cellular behaviors of iPSCs such as proliferation and differentiation potential [14–17]. Till now, multiple types of cells have been used in reprogramming, and among them, fibroblasts and astrocytes were the widely used two [8, 10, 13, 18, 22, 23]. Unlike fibroblasts, astrocytes are differentiated from NPCs and resident in the CNS. Thus, astrocytes retain a “memory” of their tissue origin and have high neurogenic competence after reprogramming, confirmed by our previous and this study [17]. More importantly, astrocytes, the most common type of cells in the CNS, get activated and start to proliferate in response to brain injury, making them an excellent source for the in vivo reprogramming [24–26]. Our observations suggested that, although both FiNPCs and AiNPCs have high proliferative capacities, FiNPCs generated more neurosphere, exhibited subtly but significantly higher proportions of Ki67^+^ cell, and expressed higher levels of *Ki67* transcripts, compared with AiNPCs, revealing a higher proliferation potential. More importantly, our data demonstrated that, similar to the situation in iPSCs, AiNPCs displayed higher neurogenic competence when compared with FiNPCs. This notion is supported by the following observations. First, AiNPCs expressed higher levels of *Sox2*, an important functional marker of NPCs [27], than FiNPCs. Second, AiNPCs exhibited higher potential to generate more glia and neurons, including different subtypes of forebrain neurons, versus FiNPCs. Third, AiNPCs exhibited higher capacities in mobility and survival, two key attributes which help transplanted iNPCs to migrate to injury sites and replace degenerating cells. Lastly, whole transcriptome analysis identified the activation of key signaling in promoting neurogenesis (e.g. TGFβ and Hippo signaling) and the inhibition of ND-related signaling in AiNPCs. Interestingly, we observed that, though AiNPCs have higher differentiation potential for both neuronal and glial lineages, the difference in the GFAP^+^ cells between AiNPCs and FiNPCs is greater than that of Tuj1^+^ cells. It could be due to the cell origins of iNPCs. For instance, the reprogrammed astrocytes may retain the “glial memory” that favors them more towards neuroglial fate than neuronal one under differentiation conditions, while reprogrammed fibroblasts lack these innate programs to push them into neither fate.

It is worth-noting that multiple strategies have been used for examining the differentiation potential of NPCs (and NPC-like cells), which can be divided into two classes. The first one uses universal conditions to differentiate NPCs into both neurons and glia, which provides a more unambiguous observation for the cell fate commitment between neuronal and glial lineages [28–30]. However, oligodendrocytes are always missing as NPCs spontaneous differentiation conditions only generate neurons and astrocytes. Another class of approaches is to induce the generation of neurons [31], astrocytes [31, 32], and oligodendrocytes [33] separately with distinct culture conditions, which are generally used to test the genesis of specific cell types. In order to compare both neuronal and glial differentiation capacities of iNPCs at the same time, we chose a growth factor free condition that has been constantly used in our previous studies [29, 34, 35]. And the oligodendrocyte generation potential of both iNPC lines will be examined in our future studies.

Though AiNPCs may have higher neurogenic potential than other types of reprogrammed cells, the underlying mechanisms remain largely unknown. One possible mechanism could be the genetic and epigenetic inheritance of reprogrammed cells from their donors [14, 15]. For example, AiNPCs inherit the demethylated status of CpG islands in SSEA1 promotor regions and Sox9 hyperexpression from astrocytes [10, 36, 37]. These characteristics reduce the difficulties of astrocytes to across lineage barrier in reprogramming and acquire neurogenic potential. In this study, we for the first time examined the gene expression profiles of AiNPCs and FiNPCs to identify the potential mechanisms regulating their distinct neurogenic competence, in which higher activities of TGFβ signaling was found in AiNPCs. TGFβ signaling has been shown to be tightly associated with neurogenesis [38]. The activation of TGFβ signaling in NPCs leads to less cell division, more neuronal differentiation and higher cell survival rate [39, 40]. Interestingly, TGFβ signaling could crosstalk with Hippo signaling through YAP, providing a possible mechanism for the activation of the latter in AiNPCs [41, 42]. Our data implied that Hippo signaling may also regulate the differentiation, invasive, and survival potential of AiNPCs (Additional file 1: Figure S4). However, we did not observe significant expression changes of Hippo signaling-related genes after LY2157299 treatment (Additional file 1: Figure S5), suggesting TGFβ signaling may regulate iNPCs through a Hippo signaling-independent mechanism. Furthermore, we observed the repression of ND-related signaling in AiNPCs. These unforeseen findings provided a new perspective to interpret the advantages in utilizing astrocytes for reprogramming.

Another interesting observation is that AiNPCs exhibit significantly higher levels of *Sox2* expression than FiNPCs. Sox2 has been considered as a master regulator for direct reprogramming of iNPCs due to its inhibitory effects on mesendodermal differentiation and positive influence on neural ectodermal fate commitment [43]. On the other hand, Sox2 also prevents cell cycle exit and NPC’s differentiation [44, 45]. Thus, the involvement of Sox2 in regulating the neural potentials of iNPCs remains vague, which needs to be extensively investigated in our future studies.

## Conclusions

iNPCs, derived from either astrocytes or non-neural cells (e.g. fibroblasts), displayed distinct capacities in proliferation, differentiation, migration and survival. These differences were likely mediated by the coordinated regulation of TGFβ signaling. Our study provides valuable information for donor cell selection in cell-based therapy for devastating ND.

## Supplementary information

**Additional file 1: Figure S1.** Reprogramming of fibroblasts into FiNPCs. (A) The overexpression of Sox2, Brn2 and Foxg1in FiNPCs was analyzed using qPCR analysis. Data were normalized to GAPDH and presented as fold change compared with fibroblasts. (B) FiNPCs generated neurospheres with similar morphology as NPCs-derived neurospheres. (C) FiNPCs were positive for proliferation marker Ki67 and NPCs-specific markers Nestin & Sox2. (D) FiNPCs were placed in neuronal, astrocyte, and oligodendrocyte differentiation media and the generation of Tuj1^+^ neurons, GFAP^+^ astrocytes and O4^+^ oligodentrocytes was determined by immunocytochemistry. (E, F) FiNPCs were placed in neuronal differentiation media and stained with MAP2, NeuN, vGlut, GABA, ChAT, and TH. Scale bars represent 50 μm (C-F) and 100 μm (B). Error bars denote s.d. from triplicate measurements. **Figure S2.** Validation of TGFβ signaling inhibitor, LY2157299. (A) The expression levels of TGFβ signaling-relate genes in FiNPCs and AiNPCs after LY2157299 treatment were analyzed using qPCR analysis. (B, C) The phosphorylation of Smad2 in FiNPCs (B) and AiNPCs (C) after LY2157299 treatment was analyzed using western blot. qPCR data were normalized to GAPDH and presented as fold change compared with fibroblasts. Error bars denote s.d. from triplicate measurements. *P < 0.05, **P < 0.01, ***P < 0.001, and ***P < 0.0001 by two-tailed *t* test (*n* = 3). **Figure S3.** The promoter methylation rates of Tgfb3 and Tgfbr2 by BiSulfite Amplicon Sequencing analysis. (A, B) The CpG methylation of Tgfb3 and Tgfbr2 promotor regions in FiNPCs, AiNPCs, fibroblast and astrocytes was determined by BiSulfite Amplicon Sequencing and represented in fold change. Fold change of the CpG methylation ratio was given on the right panel. Error bars denote s.d. *P < 0.05, **P < 0.01 by two-tailed *t* test. **Figure S4.** Hippo signaling regulates neurogenic, migration, and survival capacity of iNPCs. (A) The transcript expression of *Ki67*, *Sox2*, and *Nestin* was determined by qPCR analysis. (B) The transcript expression of *Tuj1* and *GFAP* was determined by qPCR analysis. (C) Photographs of identical fields of cells were taken for the FiNPCs and AiNPCs groups at 0 h and 24 h in wound healing assay (left panel). The total number of invading cells of each field was counted and represented in fold change (right panel). (D) Photographs of identical fields of TUNEL^+^ cells were taken for FiNPCs and AiNPCs in differentiation conditions (left panel). The total number of TUNEL^+^ cells was counted and represented in proportions (right panel). Scale bars represent 100 μm (C, D). Data were normalized to GAPDH and presented as fold change. Error bars denote s.d. from triplicate measurements. *P < 0.05, ***P < 0.001, and ***P < 0.0001 by two-tailed *t* test (n = 3). **Figure S5.** TGFβ signaling did not regulate Hippo signaling. The transcript expression of Hippo signaling-relate transcripts, *Wwc1*, *Sav1*, *Mob1a*, *Mob1b*, and *Ppp2ca* was determined by qPCR analysis. Data were normalized to GAPDH and presented as fold change. Error bars denote s.d. from triplicate measurements. *P < 0.05, ***P < 0.001, and ***P < 0.0001 by two-tailed *t* test (n = 3).**Table S1.** List of gene specific primers. **Table S2.** List of primary antibodies

## Data Availability

The datasets used and/or analyzed during the current study are available from the corresponding authors on reasonable request.

## Abbreviations

AD: Alzheimer’s disease
AiNPCs: Astrocyte-derived induced neural progenitor cells
BPs: Biological processes
CNS: Central nervous system
DEG: Differentially expressed genes
ESCs: Embryonic stem cells
FiNPCs: Fibroblast-derived induced neural progenitor cells
FPKM: Fragments per kilobase of transcript per million fragments mapped
GO: Gene Ontology
iNPCs: Induced neural progenitor cells
iPSCs: Induced pluripotent stem cells
KEGG: Kyoto Encyclopedia of Genes and Genomes
ND: Neurodegenerative diseases
NPCM: NPC favoring medium
NPCs: Neural progenitor cells
PD: Parkinson’s disease
PFA: Paraformaldehyde
qPCR: Quantitative polymerase chain reaction
RT: Room temperature
T3: 3, 3, 5-triiodothyronine
