# Supplementary material for: Reprogrammed astrocytes display higher neurogenic competence, migration ability and cell death resistance than reprogrammed fibroblasts
Source: Transl Neurodegener. 2020 Feb 8;9:6. doi: 10.1186/s40035-020-0184-6 (PMC7011554; doi:10.1186/s40035-020-0184-6)
Supplement: Supplementary file 1 — Additional file 1: Figure S1. Reprogramming of fibroblasts into FiNPCs. (A) The overexpression of Sox2, Brn2 and Foxg1in FiNPCs was analyzed using qPCR analysis. Data were normalized to GAPDH and presented as fold change compared with fibroblasts. (B) FiNPCs generated neurospheres with similar morphology as NPCs-derived neurospheres. (C) FiNPCs were positive for proliferation marker Ki67 and NPCs-specific markers Nestin & Sox2. (D) FiNPCs were placed in neuronal, astrocyte, and oligodendrocyte differentiation media and the generation of Tuj1+ neurons, GFAP+ astrocytes and O4+ oligodentrocytes was determined by immunocytochemistry. (E, F) FiNPCs were placed in neuronal differentiation media and stained with MAP2, NeuN, vGlut, GABA, ChAT, and TH. Scale bars represent 50 μm (C-F) and 100 μm (B). Error bars denote s.d. from triplicate measurements. Figure S2. Validation of TGFβ signaling inhibitor, LY2157299. (A) The expression levels of TGFβ signaling-relate genes in FiNPCs and AiNPCs after LY2157299 treatment were analyzed using qPCR analysis. (B, C) The phosphorylation of Smad2 in FiNPCs (B) and AiNPCs (C) after LY2157299 treatment was analyzed using western blot. qPCR data were normalized to GAPDH and presented as fold change compared with fibroblasts. Error bars denote s.d. from triplicate measurements. *P < 0.05, **P < 0.01, ***P < 0.001, and ***P < 0.0001 by two-tailed t test (n = 3). Figure S3. The promoter methylation rates of Tgfb3 and Tgfbr2 by BiSulfite Amplicon Sequencing analysis. (A, B) The CpG methylation of Tgfb3 and Tgfbr2 promotor regions in FiNPCs, AiNPCs, fibroblast and astrocytes was determined by BiSulfite Amplicon Sequencing and represented in fold change. Fold change of the CpG methylation ratio was given on the right panel. Error bars denote s.d. *P < 0.05, **P < 0.01 by two-tailed t test. Figure S4. Hippo signaling regulates neurogenic, migration, and survival capacity of iNPCs. (A) The transcript expression of Ki67, Sox2, and Nestin was deter [file 40035_2020_184_MOESM1_ESM.docx]

**Supplemental Information**

**Reprogrammed astrocytes display higher neurogenic competence, migration ability and cell death resistance than reprogrammed fibroblast**

Xiaohuan Xia, Chunhong Li, Yi Wang, Xiaopei Deng, Yizhao Ma, Jialin C. Zheng

**Supplemental Figures and Legends**

**
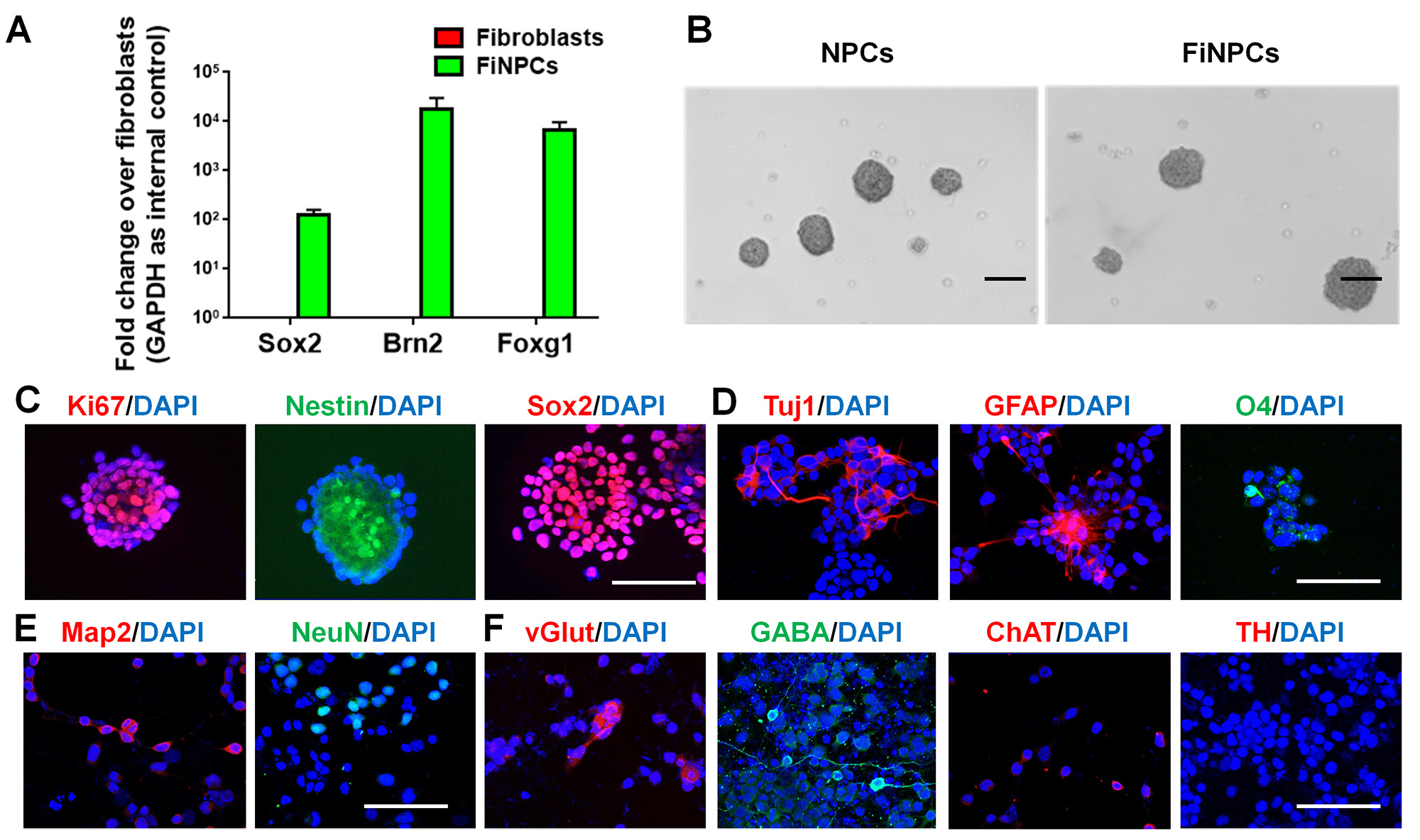
**

**Figure S1. Reprogramming of fibroblasts into FiNPCs.**

(**A**) The overexpression of Sox2, Brn2 and Foxg1in FiNPCs was analyzed using qPCR. Data were normalized to GAPDH and presented as fold change compared with fibroblasts. (**B**) FiNPCs generated neurospheres with similar morphology as NPCs-derived neurospheres. (**C**) FiNPCs were positive for proliferation marker Ki67 and NPCs-specific markers Nestin & Sox2. (**D**) FiNPCs were placed in neuronal, astrocyte, and oligodendrocyte differentiation media and the generation of Tuj1^+^ neurons, GFAP^+^ astrocytes and O4^+^ oligodentrocytes was determined by immunocytochemistry. (**E, F**) FiNPCs were placed in neuronal differentiation media and stained with MAP2, NeuN, vGlut, GABA, ChAT, and TH. Scale bars represent 50 μm (**C-F**) and 100 μm (**B**). Error bars denote s.d. from triplicate measurements.

**
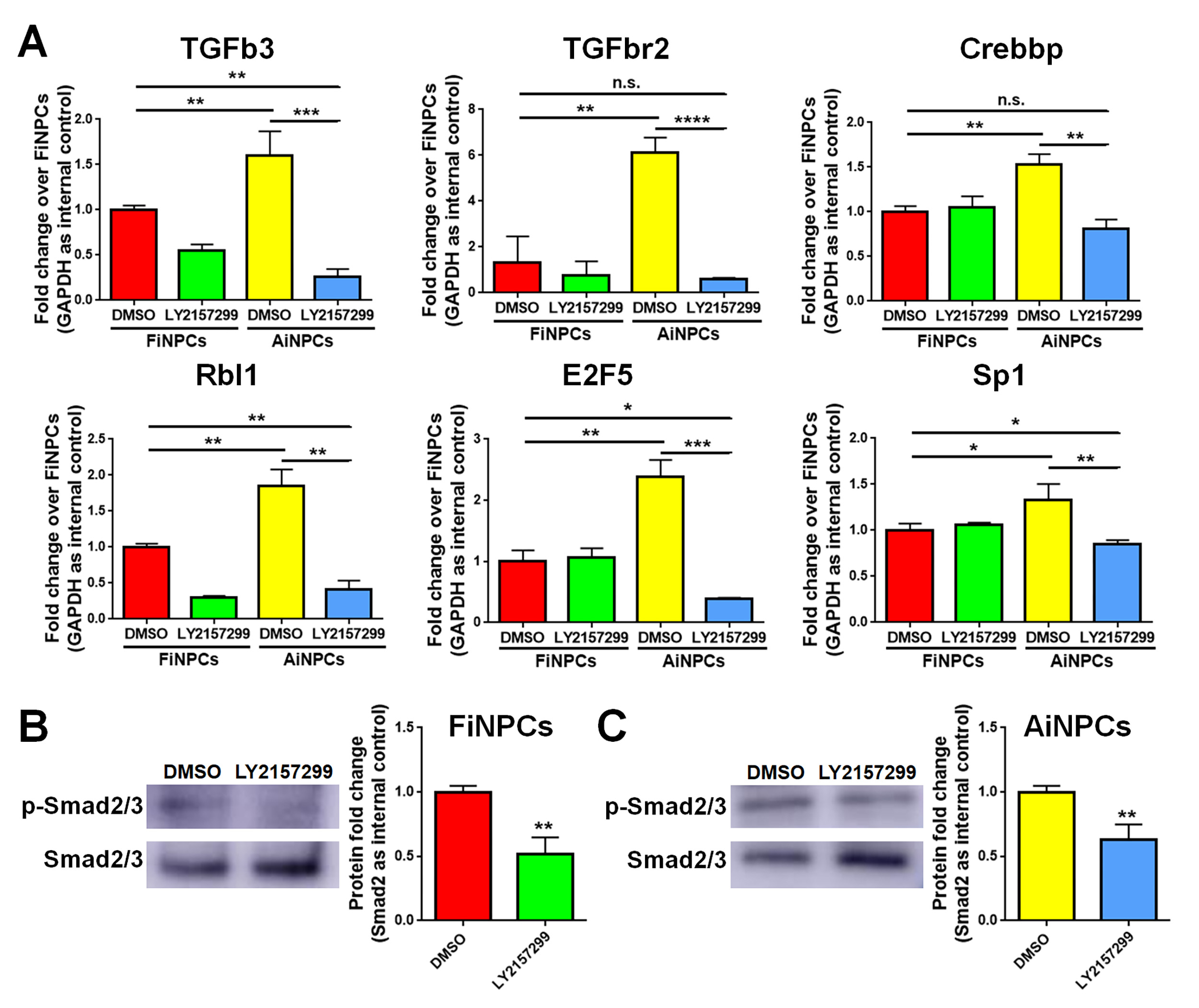
**

**Figure S2. Validation of TGFβ signaling inhibitor, LY2157299.**

(**A**) The expression levels of TGFβ signaling-relate genes in FiNPCs and AiNPCs after LY2157299 treatment were analyzed using qPCR. (**B, C**) The phosphorylation of Smad2 in FiNPCs (**B**) and AiNPCs (**C**) after LY2157299 treatment was analyzed using western blot. qPCR data were normalized to GAPDH and presented as fold change compared with fibroblasts. Error bars denote s.d. from triplicate measurements. *P<0.05, **P<0.01, ***P<0.001, and ***P<0.0001 by two-tailed *t* test (n=3).


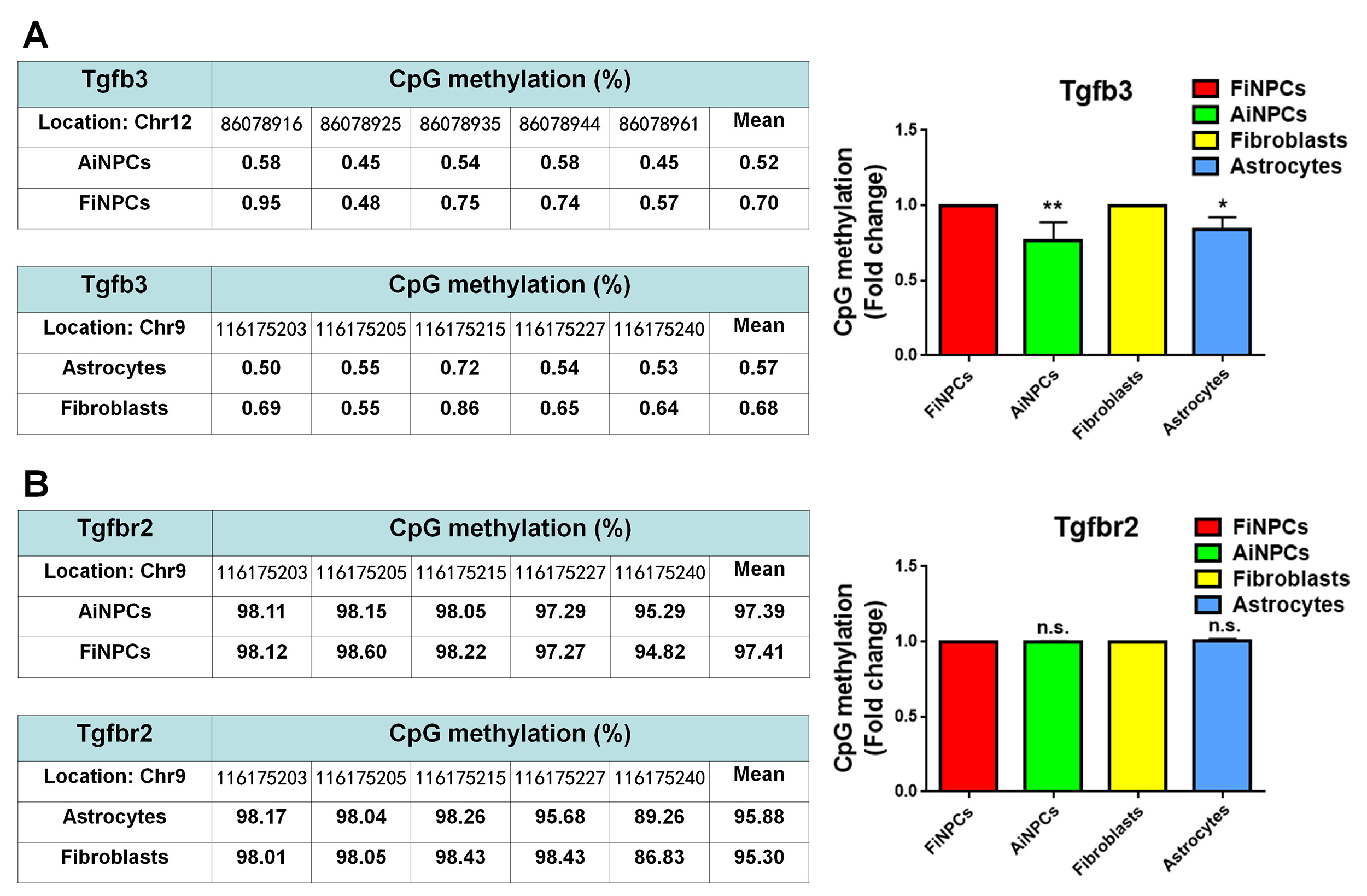


**Figure S3. Validation of TGFβ signaling inhibitor, LY2157299.**

(**A, B**) The CpG methylation of Tgfb3 and Tgfbr2 promotor regions in FiNPCs, AiNPCs, fibroblast and astrocytes was determined by BiSulfite Amplicon Sequencing and represented in fold change. Fold change of the CpG methylation ratio was given on the right panel. Error bars denote s.d. *P<0.05, **P<0.01 by two-tailed t test.


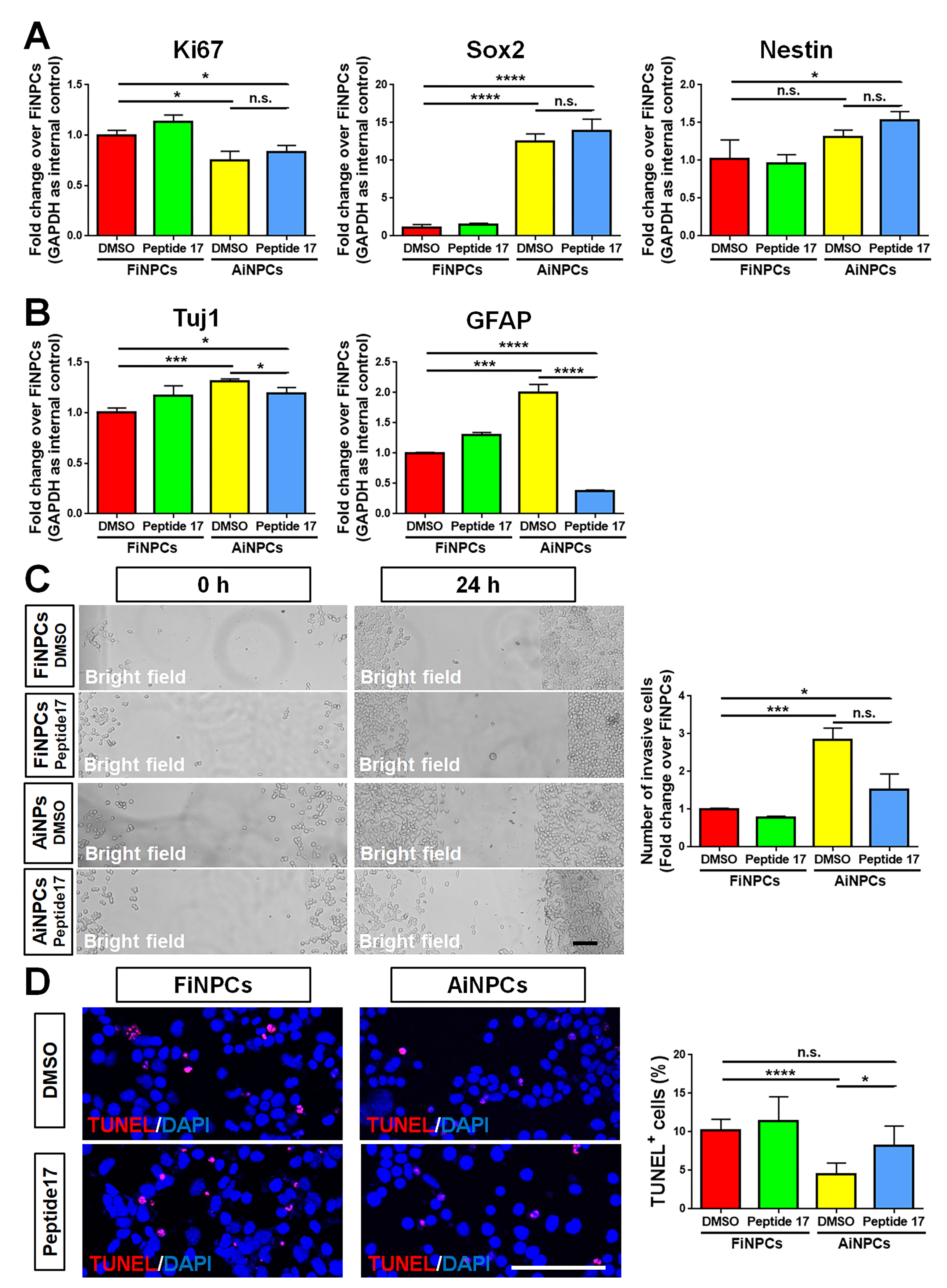


**Figure S4. Hippo signaling regulates neurogenic, migration, and survival capacity of iNPCs.**

(**A**) The transcript expression of *Ki67*, *Sox2*, and *Nestin* was determined by qPCR analysis. (**B**) The transcript expression of *Tuj1* and *GFAP* was determined by qPCR analysis. (**C**) Photographs of identical fields of cells were taken for the FiNPCs and AiNPCs groups at 0 and 24 h in wound healing assay (left panel). The total number of invading cells of each field was counted and represented in fold change (right panel). (**D**) Photographs of identical fields of TUNEL^+^ cells were taken for FiNPCs and AiNPCs in differentiation conditions (left panel). The total number of TUNEL^+^ cells was counted and represented in proportions (right panel). Scale bars represent 100 µm (**C, D**). Data were normalized to GAPDH and presented as fold change. Error bars denote s.d. from triplicate measurements. *P<0.05, **P<0.01, ***P<0.001, and ***P<0.0001 by two-tailed *t* test (n=3).


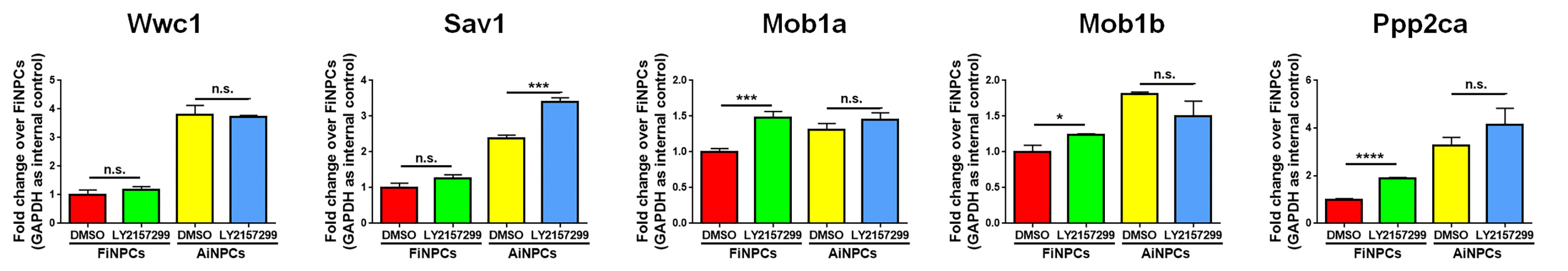


**Figure S5. TGFβ signaling did not regulate Hippo signaling.**

The transcript expression of Hippo signaling-relate transcripts, *Wwc1*, *Sav1*, *Mob1a*, *Mob1b*, and *Ppp2ca* was determined by qPCR analysis. Data were normalized to GAPDH and presented as fold change. Error bars denote s.d. from triplicate measurements. *P<0.05, ***P<0.001, and ***P<0.0001 by two-tailed *t* test (n=3).

**Table. S1. List of gene specific primers**

| Gene | Sequence | Size(bp) | T^o^ | Accession N. |
| --- | --- | --- | --- | --- |
| *Adam11* | 5’-CTGTTTGCTGTCCCTGCCCA-3’  5’-GCCGCATCTGCTCAAACAGC-3’ | 154 | 57 | NM_001110778.2 |
| *Apbb1* | 5’- GCAATGCTCGCTGCTTGGTC-3’  5’- ACTGACTCCAGGGCCCCATT-3’ | 187 | 57 | NM_001253885.1 |
| *App* | 5’- AGTAGAAGTCGCCGAAGAGG-3’  5’- CAGTGCTGGTTGTTCTCTCG-3’ | 143 | 57 | NM_001198823.1 |
| *Appbp2* | 5’-CCATCTCCGCTGTCGTGGAC-3’  5’-GCTGACACAAGCGTCCCTGT-3’ | 117 | 57 | NM_025825.3 |
| *Bace1* | 5’-TACCTGTGGCCAGAGCACCT-3’  5’-CACATGCCGGGTAGCCTGAG-3’ | 299 | 57 | NM_011792.6 |
| *Crebbp* | 5’-CTACAGCAGCGCCTCCTTCC-3’  5’-CAGGTTCTCCATGCGGCGAT-3’ | 155 | 58 | NM_001025432.1 |
| *E2F5* | 5’-AGCCTGGGCTTGCTTACCAC-3’  5’-GCCAGCACCTACACCCTTCC-3’ | 201 | 57 | NM_007892.2 |
| *Fas* | 5’-TCTGGTGCTTGCTGGCTCAC-3’  5’-TTCCCTTCTGTGCATGGGGC-3’ | 228 | 57 | NM_001146708.1 |
| *Gapdh* | 5’-CATGTTCCAGTATGACTCCACTC-3’  5’-GGCCTCACCCCATTTGATGT-3’ | 136 | 60 | NM_001289726.1 |
| *Ki67* | 5’-CAGCAGAAGAATCGTGGGAGAC-3’  5’-CCTACTTTGGGTGAAGAGGTTGC-3’ | 103 | 54 | NM_001081117.2 |
| *Mob1a* | 5’-GCGACTCTCGGAAGTGGCAA-3’  5’-GACCTGCCGACATGACTGGG- 3’ | 172 | 57 | NM_145571.2 |
| *Mob1b* | 5’-GGCACCACTGCAAGAGCTGA-3’  5’-AAGGGAACCCAAGCAAGCCC- 3’ | 173 | 57 | NM_026735.2 |
| *Nestin* | 5’-GGAGAGTCGCTTAGAGGTGC-3’  5’-TCAGGAAAGCCAAGAGAAGC-3’ | 327 | 57 | NM_016701.3 |
| *Park2* | 5’-TGGTGCTGGAACTGTGGCTG-3’  5’-CTGTGGGTGCCCTGGAAAGG-3’ | 237 | 57 | NM_001317726.1 |
| *Pink1* | 5’-GGACCGCTACCGCTTCTTCC-3’  5’-CTTCCGCCTGCTTCTCCTCG-3’ | 161 | 57 | NM_026880.2 |
| *Ppp2ca* | 5’-TCCGAGCACTCGATCGCCTA-3’  5’-GCTGGTGAGCTCTGGACACC-3’ | 189 | 57 | NM_019411.4 |
| *Rbl1* | 5’-GCTCACCCCGTACTTTCCCG-3’  5’-AGAGCCTCCCACAGTGCAGA-3’ | 193 | 57 | NM_001139516.1 |
| *Sav1* | 5’- CAGCTGGCTGACCTGGACAC-3’  5’- ACCACTGCTGCCTCTGCTTG-3’ | 142 | 57 | NM_022028.2 |
| *Snca* | 5’-GTGGCAGAGGCAGCTGGAAA-3’  5’-ACCCTTGCCCATCTGGTCCT-3’ | 234 | 57 | NM_001042451.2 |
| *Sox2* | 5’-AGTCTCCAAGCGACGAAAAA-3’  5’-GCAAGAAGCCTCTCCTTGAA-3’ | 141 | 57 | NM_003106 |
| *Tgfb3* | 5’-AGAGGGCCCTGGACACCAAT-3’  5’-CTGCGGAGGTATGGGCAAGG-3’ | 163 | 57 | NM_009368.3 |
| *Tgfbr2* | 5’-TGCCGCTTCTCCCAAGTGTG-3’  5’-TCGGGACTGCTGGTGGTGTA-3’ | 132 | 57 | NM_009371.3 |
| *Uchl1* | 5’-ATCCACGCAGTGGCCAACAA-3’  5’-AAGGGCATTCGCCCATCGAG-3’ | 257 | 57 | NM_011670.2 |
| *Wwc1* | 5’-GCGAGAATGAGGCAGTCGCT-3’  5’-CACCCACCCTCCGGTCCTTA-3’ | 174 | 57 | NM_170779.2 |

**Table. S2. List of primary antibodies**

| Name | Species | Dilution | Company |
| --- | --- | --- | --- |
| ChAT | Goat | 1:500 | Millipore |
| GABA | Rabbit | 1:5000 | Sigma |
| GFAP | Rabbit | 1:500 | DAKO |
| Ki67 | Rabbit | 1:500 | Cell Signaling |
| Map2 | Rabbit | 1:2000 | Covance |
| Nestin | Chicken | 1:5000 | Novus |
| NeuN | Mouse | 1:1000 | Millipore |
| Sox2 | Rabbit | 1:500 | Abcam |
| TH | Rabbit | 1:2000 | Cal Biochem |
| Tuj1 | Rabbit | 1:500 | Sigma |
| vGlut | Rabbit | 1:1000 | SYnaptic SYstems |
